# Supplementary material for: Out-of-equilibrium processes in crystallization of organic-inorganic perovskites during spin coating
Source: Nat Commun. 2021 Sep 24;12:5624. doi: 10.1038/s41467-021-25898-5 (PMC8463609; doi:10.1038/s41467-021-25898-5)
Supplement: Supplementary file 1 — Supporting Information for publication [file 41467_2021_25898_MOESM1_ESM.pdf]

Supporting Information

Out-of-equilibrium processes in crystallization of organic-inorganic perovskites during spin coating

Shambhavi Pratap,[1,2] *Finn Babbe,*[3] *Nicola S. Barchi,*[4,5] *Zhenghao Yuan,*[4,6] *Tina Luong,*[4] *Zach Haber,*[4] *Tze-Bin Song,*[4] *Jonathan L. Slack,*[2] *Camelia V. Stan,*[2,7] *Nobumichi Tamura,*[2] *Carolin M. Sutter-Fella*[4,8*] *and Peter Müller-Buschbaum*[1,9,*]

1 Physik-Department, Lehrstuhl für Funktionelle Materialien, Technische Universität München, James-Franck-Straße 1, 85748 Garching, Germany

2 Advanced Light Source, Lawrence Berkeley National Laboratory, 1 Cyclotron Road, 94720 Berkeley, USA

3 Chemical Science Division, Joint Center for Artificial Photosynthesis, Lawrence Berkeley National Laboratory, 1 Cyclotron Road, 94720 Berkeley, USA

4 Chemical Sciences Division, Lawrence Berkeley National Laboratory, 1 Cyclotron Road, 94720 Berkeley, USA

5 Laboratoire des Matériaux Semiconducteurs, École Polytechnique Fédérale de Lausanne, CH-1015 Lausanne, Switzerland

6 Department of Chemistry, The Pennsylvania State University, University Park, 16802 Pennsylvania USA

7 NIF & Photon Science, Lawrence Livermore National Laboratory, 7000 East Ave, 94550 Livermore, USA

8 Molecular Foundry, Lawrence Berkeley National Laboratory, 1 Cyclotron Road, 94720 Berkeley, USA

9 Heinz Maier-Leibnitz-Zentrum, Technische Universität München, Lichtenbergstraße 1, 85748 Garching, Germany

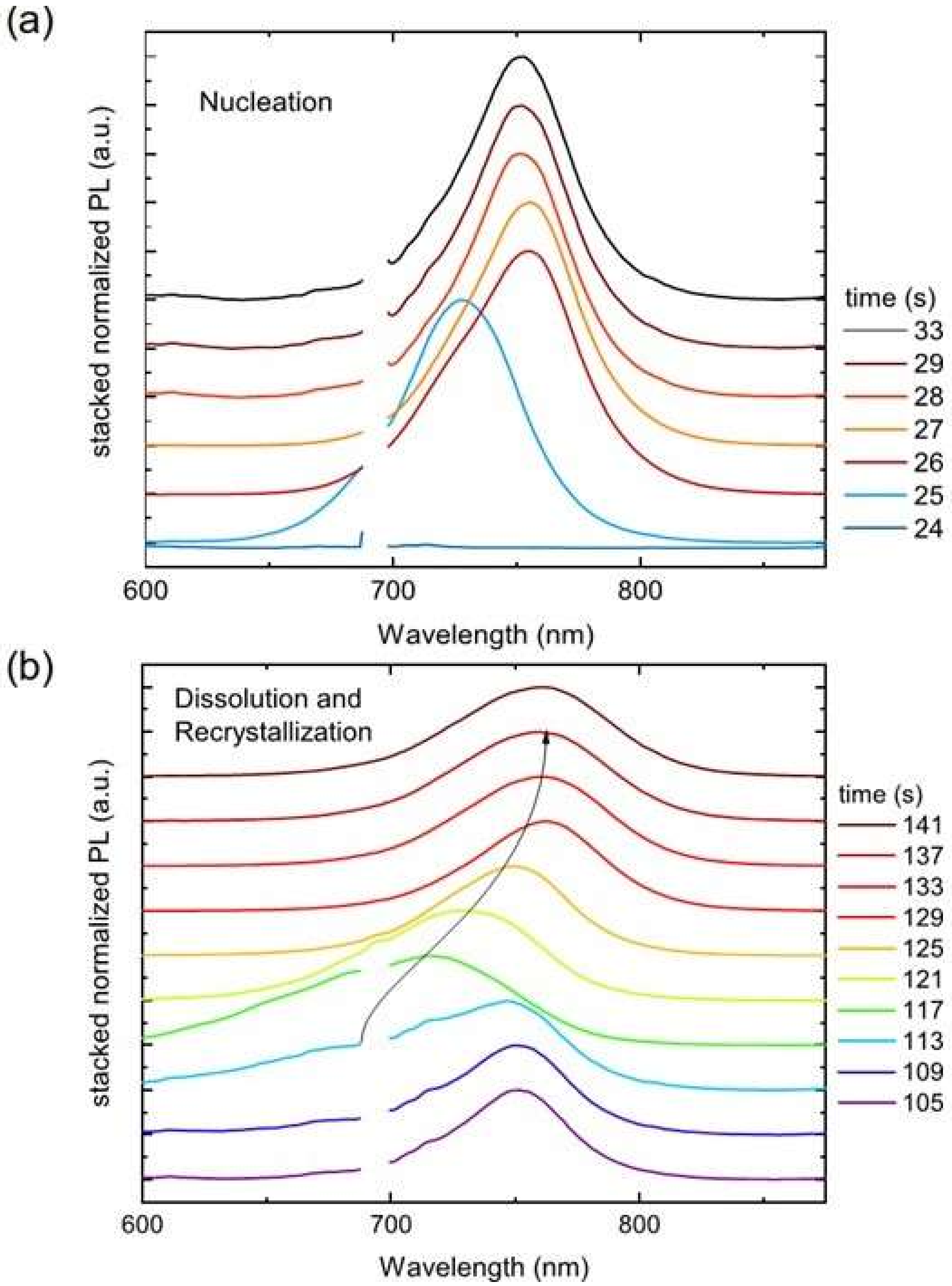

Figure S1. Temporal evolution of PL data. Normalized PL data during (a) nucleation and cluster coalescence of phase I after antisolvent drop and (b) during thermal disassociation of MAPI·DMSO solvent-complex and crystallization of MAPI on thermal annealing at 100°C. Data are stacked along the y axis with increasing time.

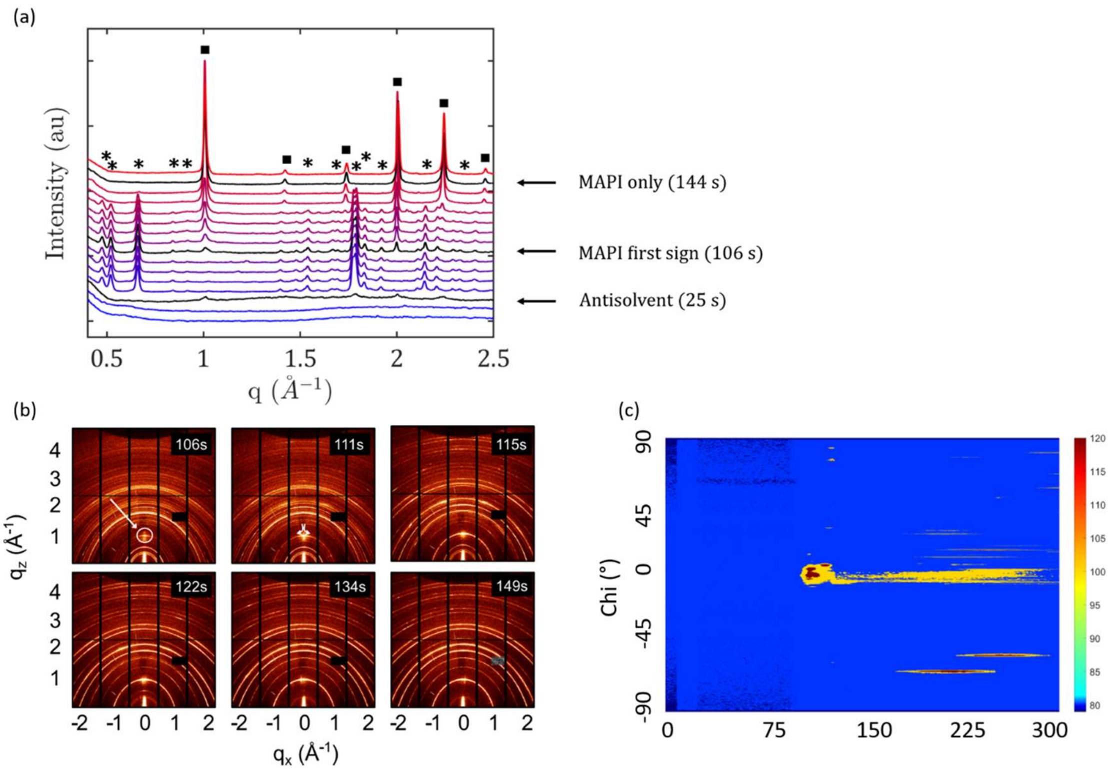

Figure S2. Temporal evolution of characteristic scattering features. (a) Selection of radially integrated 2D GIWAXS data for different stages of the *in situ* study. Characteristic stages in perovskite thin film formation are highlighted. Stars signify reflections from orthorhombic solvent-complex and filled squares signify the cubic perovskite phase. (b) 2D GIWAXS data showcasing the growth of the $MAPI_{100}$ plane at q ~ 1.0 Å^{-1} with initial face-on preferred orientation (106 s), with other crystalline orientations growing with increased annealing (111 s). Corresponding decrease of intensity from Bragg peaks of MAPI·DMSO solvent-complex at q ~ 0.7 Å^{-1}, with orientations away from the normal, lose intensity faster (115, 134 s) until complete solvent removal (149 s). (c) Evolution of distribution about azimuthal peak intensity for q ~ 1.0 Å^{-1} of the $MAPI_{100}$ plane showcasing increasing mosaic nature of the lattice preferred orientation with time.

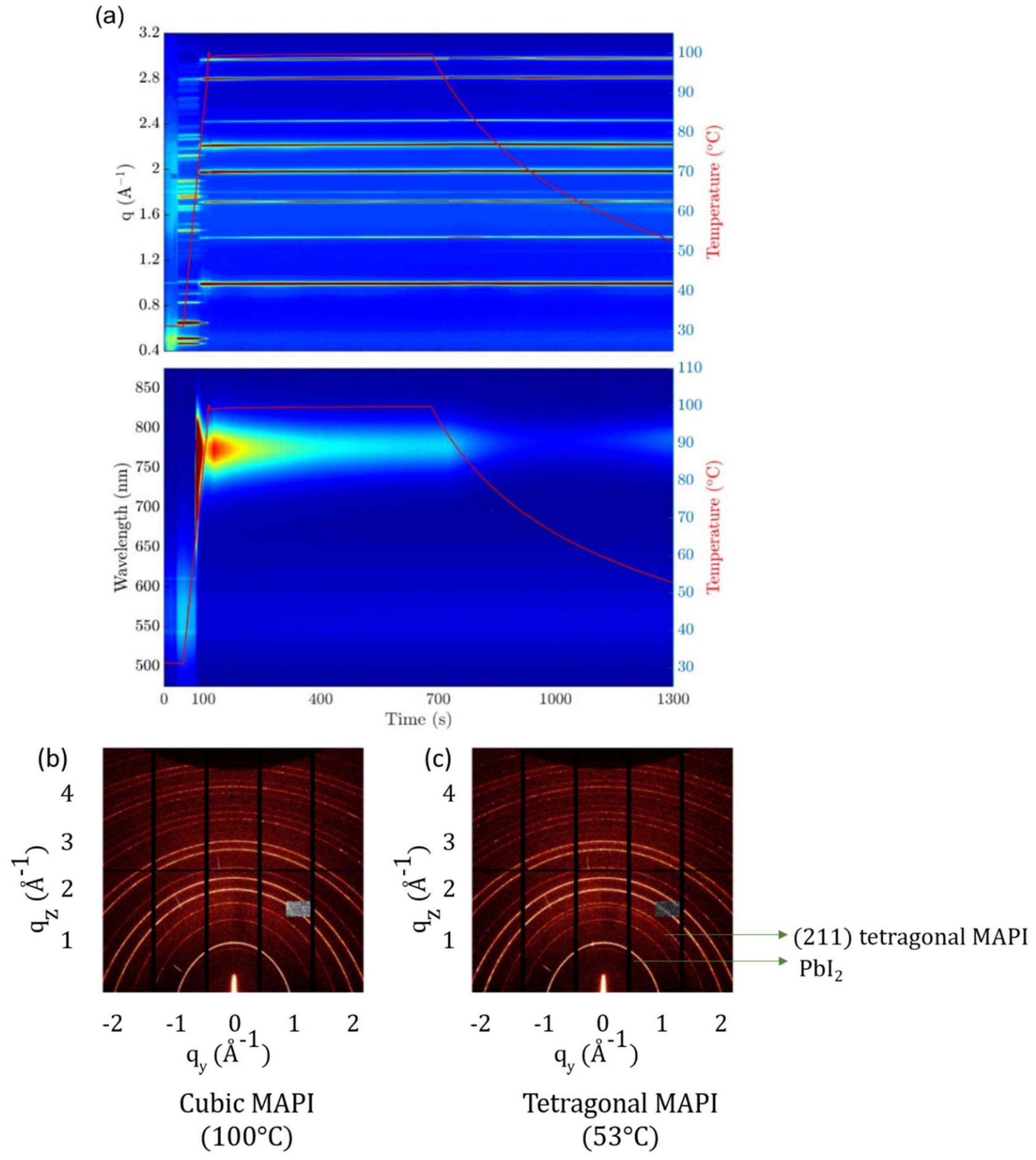

Figure S3. Long-term annealing and cooling. (a) Evolution of processing-structure-property relationship of MAPI via spinodal decomposition process via antisolvent drop, annealing and cooling. 2D GIWAXS data of (b) thin film showcasing thermal degradation of MAPI by formation of PbI_2 and (c) the transformation to tetragonal MAPI on cooling.

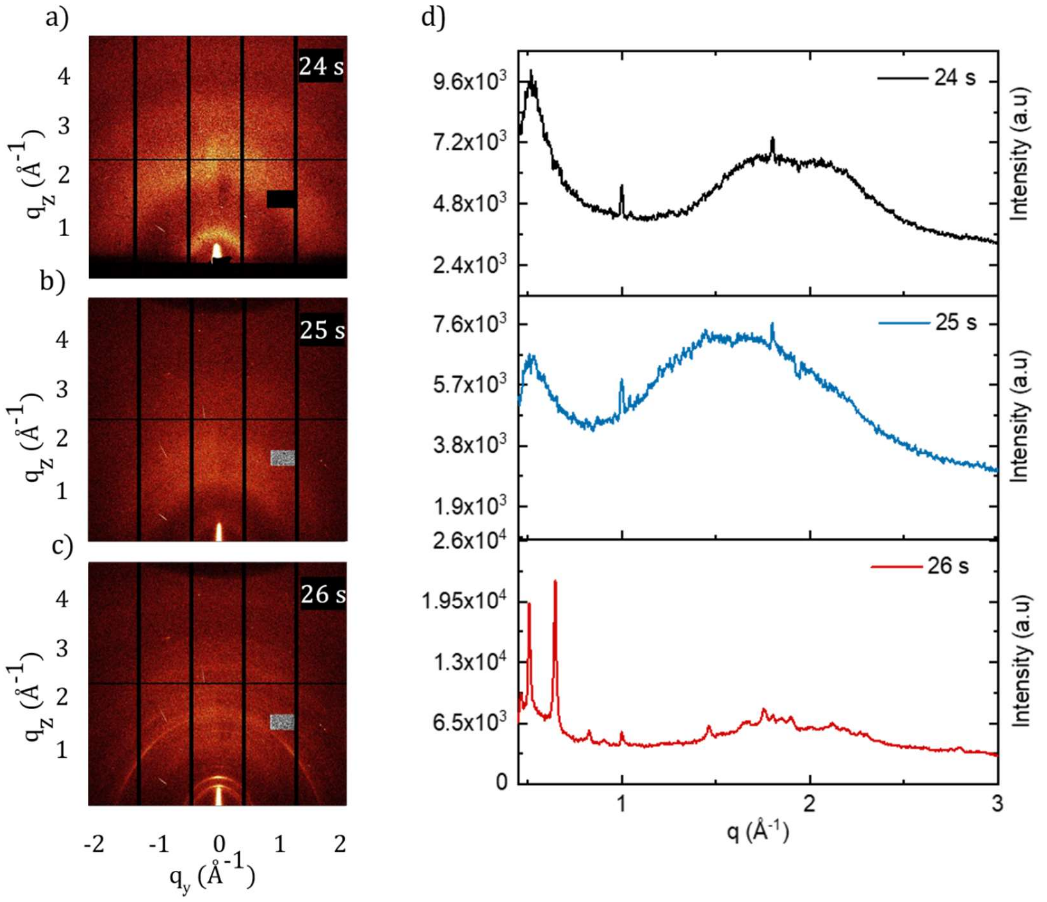

Figure S4. Crystallization by fluctuating solvodynamics. 2D GIWAXS data showing crystallization events of (a) the precursor colloid prior to the drop of the antisolvent, (b) solvodynamic, long-wavelength fluctuations in the precursor causing in changes in the colloidal background intensity, (c) resulting in the direct formation of MAPI·DMSO solvent complexes. (d) Corresponding radially integrated 2D GIWAXS data of the events involved during MAPI crystallization by spinodal decomposition
